# Synergistic Impacts of Organic Acids and pH on Growth of *Pseudomonas aeruginosa*: A Comparison of Parametric and Bayesian Non-parametric Methods to Model Growth

**DOI:** 10.3389/fmicb.2018.03196

**Published:** 2019-01-08

**Authors:** Francesca M. L. Bushell, Peter D. Tonner, Sara Jabbari, Amy K. Schmid, Peter A. Lund

**Affiliations:** ^1^School of Biosciences, University of Birmingham, Birmingham, United Kingdom; ^2^Institute of Microbiology and Infection, University of Birmingham, Birmingham, United Kingdom; ^3^Department of Biology, Duke University, Durham, NC, United States; ^4^Statistical Engineering Division, National Institute of Standards and Technology, Gaithersburg, MD, United States; ^5^School of Mathematics, University of Birmingham, Birmingham, United Kingdom; ^6^Center for Genomics and Computational Biology, Duke University, Durham, NC, United States

**Keywords:** organic acid, Gaussian process, parametric (model-based) analysis, *Pseudomonas aeruginosa*, low pH, opportunistic pathogen

## Abstract

Different weak organic acids have significant potential as topical treatments for wounds infected by opportunistic pathogens that are recalcitrant to standard treatments. These acids have long been used as bacteriostatic compounds in the food industry, and in some cases are already being used in the clinic. The effects of different organic acids vary with pH, concentration, and the specific organic acid used, but no studies to date on any opportunistic pathogens have examined the detailed interactions between these key variables in a controlled and systematic way. We have therefore comprehensively evaluated the effects of several different weak organic acids on growth of the opportunistic pathogen *Pseudomonas aeruginosa*. We used a semi-automated plate reader to generate growth profiles for two different strains (model laboratory strain PAO1 and clinical isolate PA1054 from a hospital burns unit) in a range of organic acids at different concentrations and pH, with a high level of replication for a total of 162,960 data points. We then compared two different modeling approaches for the interpretation of this time-resolved dataset: parametric logistic regression (with or without a component to include lag phase) vs. non-parametric Gaussian process (GP) regression. Because GP makes no prior assumptions about the nature of the growth, this method proved to be superior in cases where growth did not follow a standard sigmoid functional form, as is common when bacteria grow under stress. Acetic, propionic and butyric acids were all more detrimental to growth than the other acids tested, and although PA1054 grew better than PAO1 under non-stress conditions, this difference largely disappeared as the levels of stress increased. As expected from knowledge of how organic acids behave, their effect was significantly enhanced in combination with low pH, with this interaction being greatest in the case of propionic acid. Our approach lends itself to the characterization of combinatorial interactions between stressors, especially in cases where their impacts on growth render logistic growth models unsuitable.

## Introduction

Although bacterial stress responses are affected by the precise nature of the stress and the environment in which it occurs, detailed studies on the impact of systematically varying the factors that affect stress responses are rarely reported. While this is understandable from a pragmatic point of view, as varying parameters in several different factors requires multiple replicated experiments, it limits our understanding of these responses, and may mean that the conditions chosen under which to study them are not the most informative. Importantly, it also means that in many cases we lack both data and robust analytical tools that could reveal interactions between different factors, and how these might influence the ability of the organism to survive or grow in the stressful environment. This information is valuable both because it can lead to greater insights into the mechanism of the stress, and because it can have applied implications in (for example) optimizing strain construction and growth conditions for biotechnological processes, or designing effective strategies for removal of a particular organism or organisms. This latter point is particularly relevant given the pressing need to develop methods to reduce the use of antibiotics, and to treat organisms which may be resistant to multiple antibiotics.

A good example of infections which are difficult to treat are the opportunistic infections that can arise in open wounds, such as burns or skin ulcers. These often become colonized by bacteria or fungi that normally live harmlessly on the skin. Such infections are often hard to treat with antibiotics, particularly once they develop as biofilms. The two major organisms found in such infections are *Pseudomonas aeruginosa* and *Staphylococcus aureus*. The latter is the most common organism causing infection in burns, but infections caused by gram negative bacteria are the leading cause of mortality in burns patients (Branski et al., 2009; Williams et al., 2009; Høiby et al., 2010; Norbury et al., 2016). The increasing prevalence of bacteria resistant to multiple antibiotics in burns infections is associated with more surgical intervention and longer hospital stays for patients (van Langeveld et al., 2017). For this reason, alternative topical treatments are being sought for clinical use.

Organic acids (OAs) are generally more effective (at the same pH) than inorganic acids at preventing bacterial growth (Wolin, 1969; Cherrington et al., 1991; Hirshfield et al., 2003). This has led to their extensive use as preservatives in the food industry (Brul and Coote, 1999), and also to their increased use as topical agents to treat infected wounds, particularly burn wounds and diabetic ulcers (Phillips et al., 1968; Milner, 1992; Sloss et al., 1993; Nagoba et al., 2013b; Bjarnsholt et al., 2015; Halstead et al., 2015; Agrawal et al., 2017). Indeed, the use of vinegar (acetic acid) for its antibacterial properties has good historical precedents (Johnston and Gaas, 2006). The antibacterial action of OAs stems in part from their ability to diffuse freely across bacterial membranes in their uncharged state, and to then dissociate inside the bacterial cell (Salmond et al., 1984; Russell and Diez-Gonzalez, 1998; Hirshfield et al., 2003; Saparov et al., 2006; Slonczewski et al., 2009). Entry of OAs causes the proton gradient to partially collapse across the membrane, since the anion can combine with protons pumped out of the cell by the electron transport chain, then carry them back into the cell, bypassing the FoF1 ATPase (Sheu et al., 1975; Salmond et al., 1984). OAs also decrease the internal pH of the cell, although OAs are not chemiosmotic gradient uncouplers in the classical sense (Russell, 1992; Russell and Diez-Gonzalez, 1998). However, despite these effects, the toxicity of OAs does not correlate particularly well with their pKa (Sheu et al., 1975), suggesting that OA effects cannot be attributed solely to their impact on the proton gradient or the cell's internal pH. OA types vary depending on their structure in the extent to which they partition into the membrane, and the anions released when the OAs dissociate inside the cell may have different effects on cellular osmolarity and metabolism (Roe et al., 1998, 2002). The effect of OAs on growth of a given bacterial species can also vary with the particular strain chosen (King et al., 2010). Determining which OAs will be most effective against a particular bacterium therefore requires a systematic study on the combined impact of concentration and pH of several different OAs, and this needs to be done in more than one strain. However, such comprehensive comparisons have not been conducted to date for any bacterial species of which we are aware. As part of a larger investigation of the impact of OAs on the opportunistic pathogen *Pseudomonas aeruginosa*, we have therefore undertaken such a comparative study, investigating the combined effects of seven different OAs at three different concentrations and five different pH values for two different strains: a recent isolate from a burns unit, PA1054, and the strain PAO1, itself originally isolated from a wound but now used worldwide as a reference strain (Klockgether et al., 2010). These data then required analysis so that comparisons of the effects of the different factors and the interactions between them could be made in a statistically robust fashion.

Multifactorial studies into organismal behavior under stress require reproducible assays with easily measured outputs, the simplest of which are measurements of growth (or of a suitable proxy, such as optical density). In an ideal experiment, growth rates are monitored over time under a range of different conditions. Growth parameters are then extracted by fitting the data to a suitable model, and they are compared in order to evaluate the relative importance of the different conditions. Typically, these parameters include the length of lag phase, maximum growth rate during logarithmic phase, and final carrying capacity. The carrying capacity is the maximum population density which can be reached in a growth experiment; in a logistical model this value is approached asymptotically over time. In experiments, it is represented by the optical density of the culture when it reaches stationary phase. As long as the growth of the organism can be fitted to a logistic or modified logistic equation, this approach is feasible (Zwietering et al., 1990; Baranyi and Roberts, 1994). Difficulties arise, however, when bacterial growth departs from simple logistic growth, as may happen when cells are growing under stress as the cell's resources may be partially reallocated from growth to repair stress-mediated damage or to ameliorate or compensate for non-optimal conditions in the cell (Darnell et al., 2017; Tonner et al., 2017). There is an extensive literature, largely based in food microbiology where theoretical predictions of growth are crucial for developing safe manufacturing and storage processes, on ways in which deviations from simple logistic growth can be modeled and statistically tested [reviewed in Peleg and Corradini (2011)]. However, although these methods can be quite successful, they generally require either an underlying assumption of parametric growth, some prior knowledge of how growth conditions may perturb this, or complex fitting procedures.

An alternative solution to this problem is to use Gaussian process (GP) regression models for data fitting (Tonner et al., 2017), as these can provide fits to any functional form without needing any prior assumptions about the way in which genetic and/or environmental perturbations affect growth, but instead rely on a process of learning growth behavior directly from the data. A version of this approach (called B-GREAT) was developed and implemented for modeling and determining the statistical differences between growth of wild type vs. mutated strains of the model archaeal species *Halobacterium salinarum*. The growth of a large collection of different yeast strains was also tested. In both organisms, B-GREAT was found to outperform four well-used parametric methods under a range of stress conditions (Tonner et al., 2017). B-GREAT could also be used to recover standard growth parameters with high accuracy when the growth curves showed a good fit to logistic growth. B-GREAT accurately estimated these parameters, especially for growth data of strains subjected to stress (Tonner et al., 2017). A further development of this model (FANOVA) incorporated functional analysis of variance, thus allowing the effects of different factors on growth, including their interaction, to be incorporated within a unified modeling framework (Darnell et al., 2017).

In this study we have compared two parametric methods (fitting to a simple logistic equation with or without a term for the lag phase) with a Bayesian hierarchical GP model of phenotypes, called *phenom*, for their accuracy in fits to the growth data and in statistical comparisons of the effects of variations in OA and pH on growth. The major novelty of *phenom*, compared to the previous B-GREAT and FANOVA growth models of which it is an extension, is that it allows direct analysis of the interaction term. Our data show the superiority of the GP regression method over logistic parametric models, particularly when high levels of stress lead to growth behavior that deviates from the expected sigmoid curve. Further, our analyses of the model output demonstrates that the different OA types can be split into two clear groups on the basis of the growth detriment that they cause, while confirming a very strong interaction between OA and pH in affecting bacterial growth.

## Materials and Methods

### Strain Descriptions

Planktonic growth was measured for two strains of *Pseudomonas aeruginosa*: the widely used laboratory strain PAO1 (ATCC 15692), and *Pseudomonas aeruginosa* PA1054, a strain resistant to multiple antibiotics isolated from a patient in the burns unit at the Queen Elizabeth II hospital in Birmingham (Halstead et al., 2015).

### Determination of Effects of Organic Acids on Planktonic Growth

*P. aeruginosa* strains were grown in M9 minimal medium supplemented with 0.4% (w/v) glucose and 0.2% (w/v) cas-amino acids (Fisher Scientific). All growth media were buffered with 100 mM MOPS and 100 mM MES (Burton et al., 2010). Growth kinetics were recorded using a CLARIOstar automated microplate reader (BMG Labtech). Bacteria were grown in clear, flat-bottomed 96 well Costar plates. The edges of the plate (rows A and H and columns 1 and 12) contained sterile media and were used to provide blanks, to test sterility, and to minimize any edge effects. No evidence of cross-contamination was seen in any experiments. 10× M9 salts (which had a starting pH of 5.8) were adjusted to a pH of 7, 6.5, 6, 5.5, or 5 using sodium hydroxide or hydrochloric acid. The salts were then diluted and autoclaved and supplements were added subsequently; this did not cause any significant change in pH. Control experiments (not shown) showed that variation in optical density measurements across the plate was negligible; nevertheless, to minimize any systematic bias, the relative position of the different conditions on plates was changed with each experiment. OAs were added to a final concentration of either 0 mM (control), 5, 10, or 20 mM: at these concentrations, addition of OAs had no significant effect on the measured pH of the buffered growth media. Each experiment was repeated on at least two different days, with a minimum of three independent biological replicates each day. Growth experiments were initiated from independent overnight cultures diluted to a starting OD_600_ of 0.05, with the appropriate media making up the final volume to 200 μl in each well. The plates were incubated in the plate reader at 37°C, with 300 rpm shaking continuously and rigorous 500 rpm shaking for 10 s prior to each read to reduce clumping. Plates were covered with an evaporation resistant lid, maintaining aerobic conditions whilst minimizing evaporation. The OD_600_ was recorded automatically every 15 min for a total of 24 h.

### Modeling of Growth Curve Data: (1) Parametric Methods

The first method used a parametric approach to generate fits, *y* (*t*), to the OD data measurements to either the simple three-parameter logistic growth equation (which requires estimation of the initial OD value, *y*_0_, intrinsic growth rate, *r*, and carrying capacity, *K*):

$$y\left(t\right)=\frac{Ky_{0}\exp\left(rt\right)}{K+y_{0}\left(\exp\left(rt\right)-1\right)},$$

or to the four-parameter model of Baranyi and Roberts (1994), which incorporates a lag-time, λ, before the onset of exponential phase:

$$y(t)=\frac{Ky_{0}\exp\left(r\left(t-\lambda \right)\right)\left(1-\exp\left(-rt\right)+\exp\left(-r\left(t-\lambda \right)\right)\right)}{K+y_{0}\exp\left(r\left(t-\lambda \right)\right)-y_{0}\exp\left(-\lambda r\right)}.$$

The fitting was performed in (MATLAB., 2012) using the local minimiser *fminsearch* with the objective function given by the *L-2* norm of the absolute error vector. Fits were first performed for the logistic and Baranyi model against the original optical density data. For subsequent comparison with the *phenom* model, an appropriately scaled logistic model (scaled with initial value and log2 transform) was fitted to the data that was scaled for the *phenom* model analysis [as detailed below and in Tonner et al. (2017)]. The relative error (*L-2* norm of the absolute error scaled by the measured OD value at each data point) was used to assess goodness of fit. Failure to fit an accurate curve is reported when the maximum number of function evaluations is exceeded in MATLAB without tolerances being met (default options for *fminsearch* in MATLAB R2017b are used). Bad fits are reported when the relative error is >10 or when final carrying capacity is 1.5 times greater than the control condition of pH 7 with no added organic acid. Input data and model output are freely available at github.com/amyschmid/pseudomonas-organic-acids.

### Modeling of Growth Curve Data: (2) Phenom Method

#### Data Pre-processing

Raw growth data were preprocessed prior to *phenom* modeling as follows. Any time points with missing data were removed prior to analysis. The first 5 time points of every growth curve was removed prior to analysis due to the variability of the growth instrument at these early time points. Data were log2 transformed, then normalized by polynomial regression as described in Tonner et al. (2017), such that the log2 OD values started at zero for all time series. Preprocessed data were standardized to mean = 0 and standard deviation = 1 for input to the *phenom* model. Standardization of model output data was then reverted to the original mean and standard deviation, but kept log2 transformed, for plotting and interpretation. Raw and preprocessed data are provided in the github repository for this study at github.com/amyschmid/pseudomonas-organic-acids.

#### Phenom Combinatorial Model

Data measured as *y*(*t, p, m*) with pH *p* and molar acid concentration *m* was modeled with mean

$$\hat{y}(t,p,m)=\{\begin{array}{ll} \mu (t), & \text{if }p=7\text{and }m=0\\ \mu (t)+\alpha_{p}(t), & \text{if }p\neq 7\text{and }m=0\\ \mu (t)+\beta_{m}(t), & \text{if }p=7\text{and }m\neq 0\\ \mu (t)+\alpha_{p}(t)+\beta_{m}(t)+{\left(\text{αβ}\right)}_{p,m}(t), & \text{otherwise}, \end{array}$$

where μ(*t*) is the grand mean, α_*p*_(*t*) is the independent effect of pH, β_*m*_(*t*) is the independent effect of acid concentration, and (αβ)_*p, m*_(*t*) is the interaction of pH and acid concentration. The variable of primary interest is (αβ)_*p, m*_(*t*), as this represents the impact of pH and acid relative to the independent effects of the two variables. Observations are assumed to be corrupted with Gaussian noise: $y\left(t,p,m\right)=\hat{y}\left(t,p,m\right)+\epsilon$, $\epsilon \sim N\left(0,\sigma_{y}^{2}\right)$. This model (equation 1) corresponds to the null model, *M*_*null*_.

### Model Implementation

*phenom* is implemented using the STAN library, which provides efficient Hamiltonian Monte-Carlo sampling of the model posterior (Carpenter et al., 2017). Additional details of the *phenom* model will be published in a separate paper currently in preparation. Details of the *phenom* model and implementation are available at github.com/ptonner/phenom. *phenom* output data (carrying capacities, relmin values) are given in the Supplementary Material and at github.com/amyschmid/pseudomonas-organic-acids.

### *Post-hoc* Analysis of *Phenom* Model Output

Model output was graphed using the R package ggplot2 (Wickham, 2009). *P*-values of significance reported throughout the text were determined using unpaired, two-sided Student's *t*-tests. All R code for *post-hoc* analysis of *phenom* model output is provided in an R markdown file via the github repository for this study github.com/amyschmid/pseudomonas-organic-acids.

## Results

### Initial Analysis of Growth Curve Data

In order to determine the combined effects of pH and OA on growth of *P. aeruginosa*, we generated 1,680 independent growth curves, consisting of 162,960 data points after establishing optimal conditions for our experiments (Figure S1). The effects on planktonic growth of *P. aeruginosa* over 24 h were assessed under conditions varying by the nature of the strain, the specific OA used, the pH, and the concentration of the acid. A control with no added OA was included in every experiment to reduce the expected effects of day to day variation.

Visual inspection of growth curves showed both reduced growth and obvious deviations from normal logistic growth behavior, particularly when cells were grown under increasing levels of stress (a combination of low pH and high OA). As an example, the effects of different OAs at different pH but at the same concentration (arbitrarily chosen as 10 mM) are summarized in Figure S2 for strain PAO1. This series of graphs shows that the inhibitory effects of all the OAs at this specific concentration becomes greater as the pH of the medium decreases from 7 to 5, as expected, and that there is clear evidence of variation in the effects of the different OAs. However, these graphs also illustrate the difficulty of establishing the nature of the interactions between the different growth conditions, because of the large number of variables that were investigated. For this reason, we wished to find a way to extract simple growth parameters from the kinetic data, so that quantitative cross-condition and cross-strain comparisons could be made more easily. To do this, we turned to modeling of the growth curve data.

### Comparison of Different Parametric Methods for Modeling Growth Curve Data

**Figure 1:**
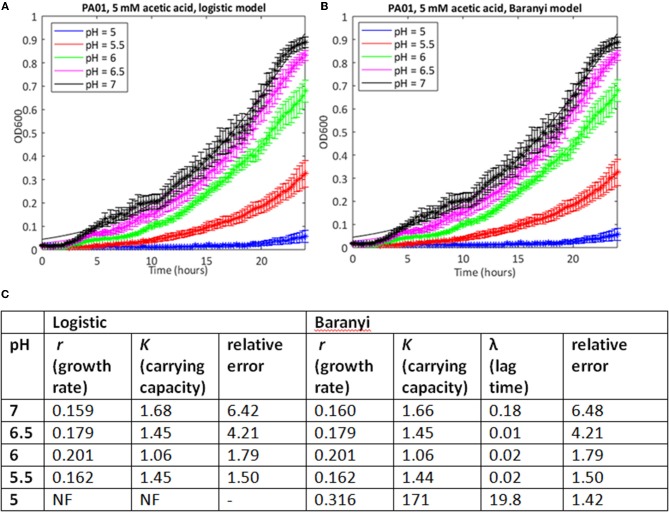
Use of a simple logistic model to fit growth curve data of strain PAO1 in 5 mM acetic acid at varying pH. Six replicates were used to generate mean and SEM values for growth over 24 h, and best fit curves (shown as continuous lines) were generated using **(A)** the logistic growth curve or **(B)** the Baranyi method, encoded in MATLAB, as described in Materials and Methods. **(C)** Model parameters and error estimates generated by the two fitting protocols. NF, no fit could be obtained. All data given to three sig. figs.

Because of the large number of growth curves generated in this study, we needed a method to estimate growth parameters where a mathematical model could be used repeatedly on a large dataset with minimal or no further input from the investigator. Initially, we used a model based on the logistic growth equation to fit the growth data, and implemented this in MATLAB. Estimates of the errors showed that in many cases, particularly where the stress level was relatively low, this approach yielded accurate fits to the observed data (Supplementary Table 1). Estimates of growth rates and carrying capacity could therefore be taken from these. As an example, the growth of PAO1 in 5 mM acetic acid at pH from 7 to 5 is shown in Figure 1A, together with growth parameter estimates, and estimates of the error in the curve fitting (see Supplementary Table 1 for data from all conditions). The data show a clear step-wise increase in the inhibitory effect of acetic acid on both growth rate and carrying capacity as the pH of the solution drops from pH 7 to 5 (data are plotted using a linear y-axis to make this difference clearer). However, this method failed to find a fit when the pH of the growth medium was reduced to 5.

**Figure 2:**
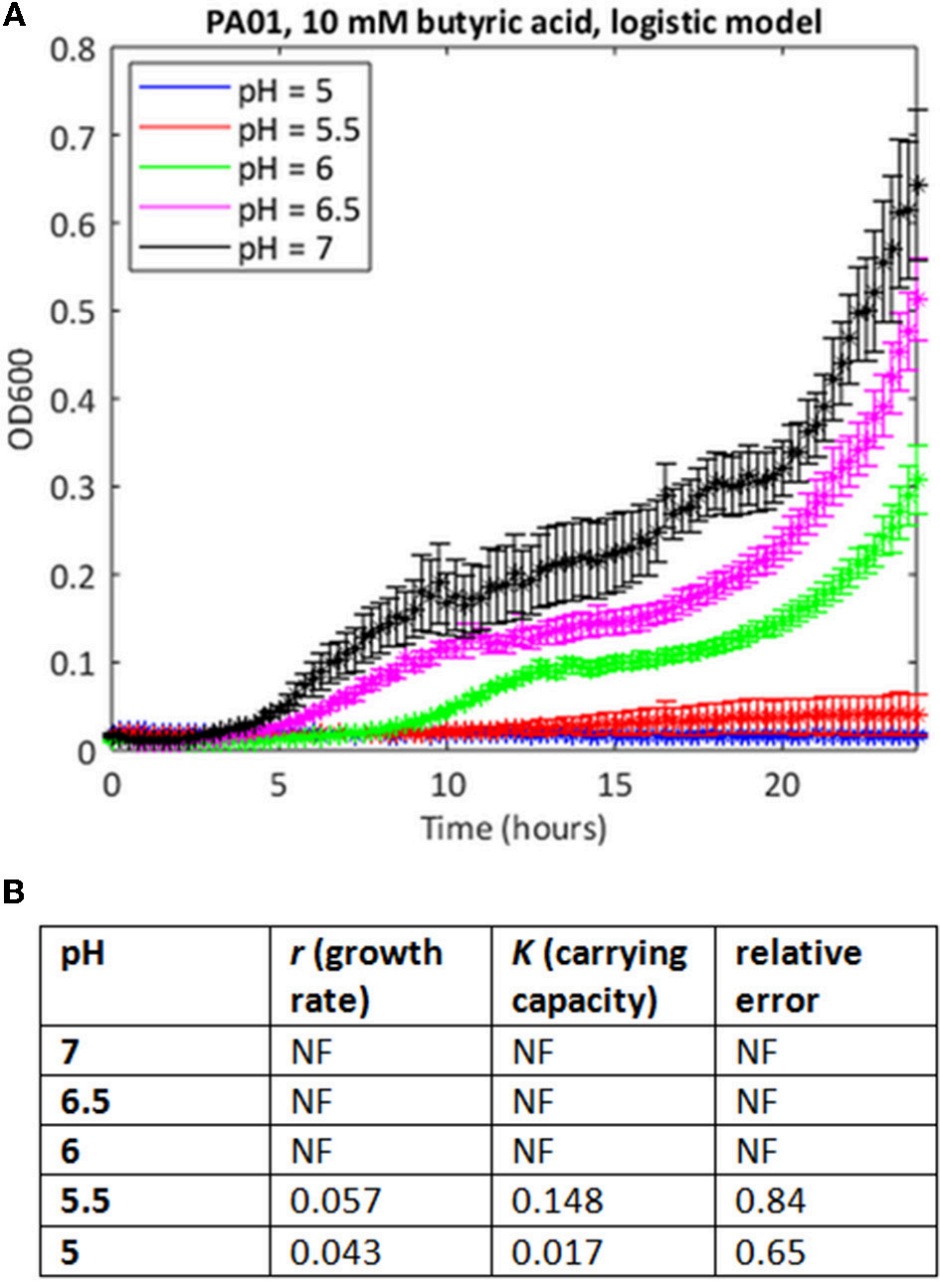
Use of the simple logistic model to fit growth curve data of strain PAO1 in 10 mM butyric acid at varying pH. **(A)** Six replicates were used to generate mean and SEM values for growth over 24 h, and best fit curves were generated as in Figure 1. If a curve could not be generated, data points are shown but there is no continuous line. **(B)** Model parameters and error estimates generated by the fitting protocol: NF, no fit could be obtained.

In many cases where the stress level was higher, the MATLAB routine failed to find a fit to the data using a logistic growth model (as defined in Materials and Methods). In some cases, model fitting failed because the data deviated from the expected logistic form of the growth curve. For example, growth in 10 mM butyric and low pH is extremely poor (Figure 2A), but at higher pH values, the growth curve deviates from the sigmoid form, exhibiting diauxie. No fit could be found for most of these data using the logistic model. In other cases, it was possible to find a fit but the estimates of growth rate (nine cases) or carrying capacities (six cases) were anomalous, being one to three orders of magnitude greater than results derived from similar but less stressful conditions. Generally, these were cases where very little growth had occurred, and where the model was overly sensitive to small increases in initial optical density in fitting the data.

**Figure 3:**
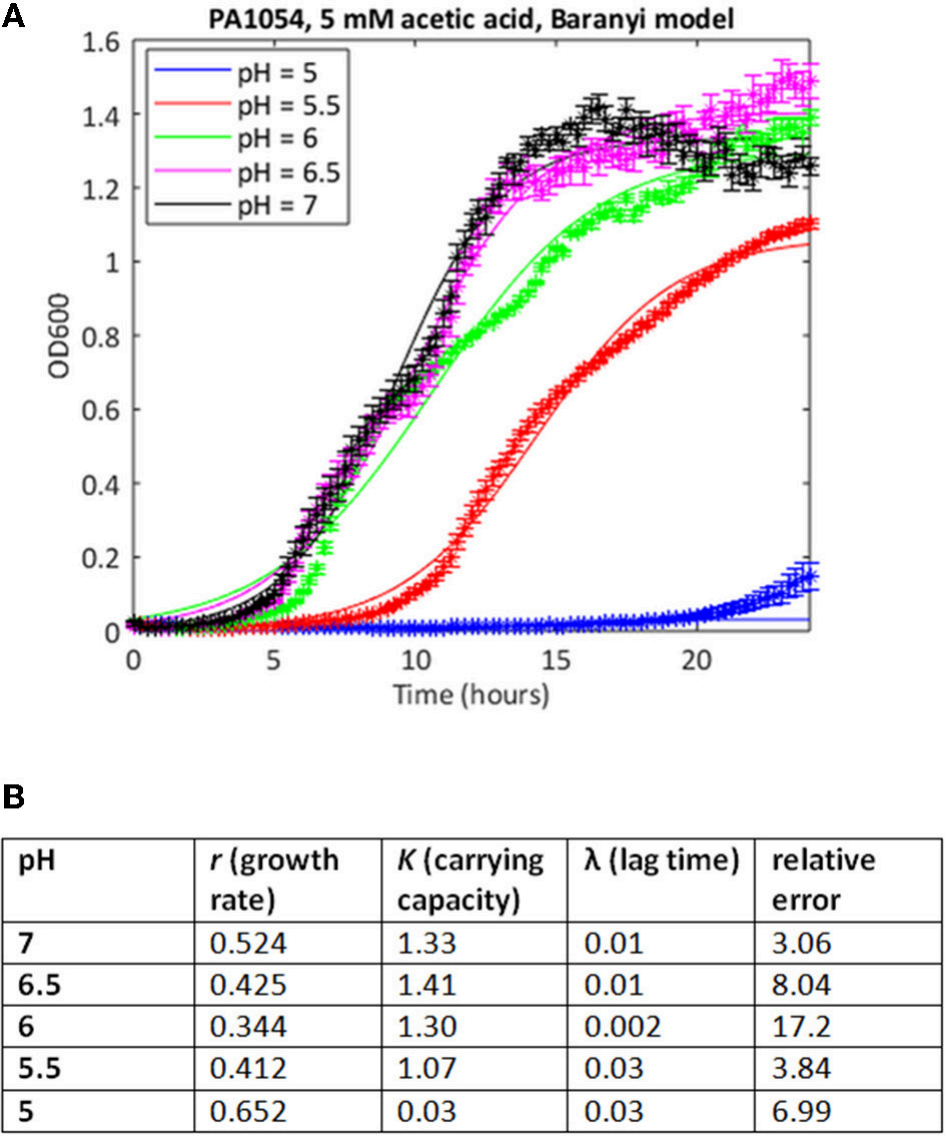
Use of Baranyi model can improve quality of curve fitting. **(A)** Growth curves of strain PA1054 grown in 5 mM acetic acid at varying pH, generated over 24 h, with best fit curves using the Baranyi growth curve model encoded in MATLAB, as described in Materials and Methods. **(B)** Model parameters and error estimates generated by the fitting protocol.

We attempted to improve the fits to the data using a Baranyi model, which accounts for a lag phase, a common feature of the growth of bacterial cultures (Baranyi and Roberts, 1994). In cases where both methods were able to find a good fit, the correlations between the parameter estimates was extremely high (Spearman's rank correlation coefficient >0.99, *p* < 0.001 for both carrying capacity and growth rate). In some cases, the Baranyi method was able to find a fit where the logistic model had failed. For example, for PA1054 growth in 5 mM acetic acid over the pH range 7 to 5, the logistic model was unable to generate a value for growth rate at pH 5 under these conditions, whereas the Baranyi model succeeded in fitting curves under all conditions (Figure 3). However, the number of failures was still high. From a total of 220 conditions tested, the logistic method failed to find a fit in a total of 28 cases (20 with PAO1, 8 with PA1054), and was considered to have generated a bad fit (as defined in Materials and Methods) in 30 additional cases (15 for PAO1 and 15 for PA1054). The Baranyi method failed in 17 cases (all PAO1), and generated a bad fit in a further 37 cases (19 PAO1 and 18 PA1054). We therefore turned to the use of the Bayesian non-parametric method *phenom* as an alternative method to estimate growth parameters.

### Non-parametric *phenom* Outperforms Parametric Logistic Growth Models for Fitting Growth Curves With Non-canonical Functional Forms

**Figure 4:**
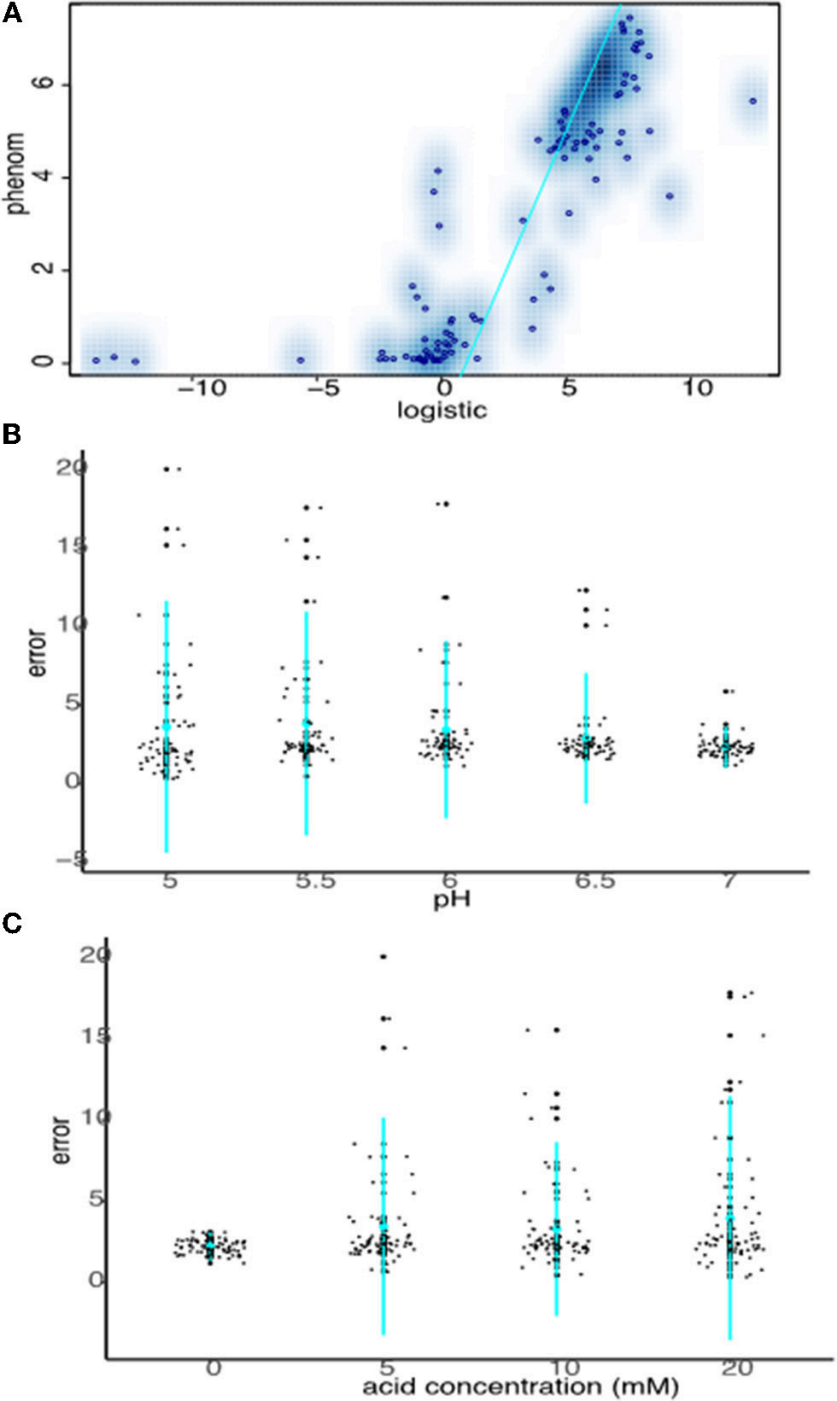
Strong carrying capacity correlation between phenom and logistic models is driven by good logistic fits under non-stress conditions. **(A)** Scatterplot comparing carrying capacity estimates of logistic model carrying capacity estimates with those from the phenom model. Individual points represent the mean of log2 carrying capacity estimates across biological replicate samples for each pH/OA condition in each strain. Blue color density estimates surrounding point clouds represents the 2D kernel density estimate obtained via the smoothScatter() function in R. Cyan line represents the linear regression of the correlation. **(B)** Comparison of logistic model error fits to the data at given pH levels across acid concentrations. Individual points represent the error fit for a given pH/OA concentration combination. Cyan points represent the mean of the data for a given pH, lines represent the standard deviation. **(C)** As in **(B)**, except errors at each OA concentration across pH levels are shown.

Data from all growth curves were fitted using the *phenom* method described in Materials and Methods, and the results were compared with estimates from the logistic method described in the previous section. We observed that carrying capacity estimates were strongly and significantly correlated between the two models (Spearman's rank ρ = 0.860, *p* ≤ 2.2e-16, Figure 4A, Supplementary Tables 1, 2). However, this correlation is primarily driven by the fact that sum of squares error estimates for the goodness-of-fit of the logistic model to the data are low (i.e., good) for pH or OA conditions alone (Figures 4B,C, Supplementary Table 1). Erroneous fits to the data from the logistic model increase substantially under pH/OA combinatorial conditions, causing a sharp drop-off in the correlation of logistic carrying capacity estimates with those from *phenom* when cultures are grown under pH and OA combined. We observed that this error inflation in the logistic model fits is due either to inaccurate fits for growth curves that deviate from the sigmoid functional form, or a complete failure to fit the data (as described above and seen in Figure 2).

Together these data demonstrate that logistic models are suitable for fitting data when growth approximates a sigmoid function with canonical lag, log, and stationary phases. However, under many of the stress conditions of interest here, which often produce non-standard or flat growth curves, the *phenom* model is more appropriate and more accurate because it is agnostic to the functional form of the data. We therefore conducted all subsequent analysis of the growth data using *phenom*.

### Growth of *P. aeruginosa* Is Significantly Impaired by Combinations of Organic Acid and pH Across Strains

**Figure 5:**
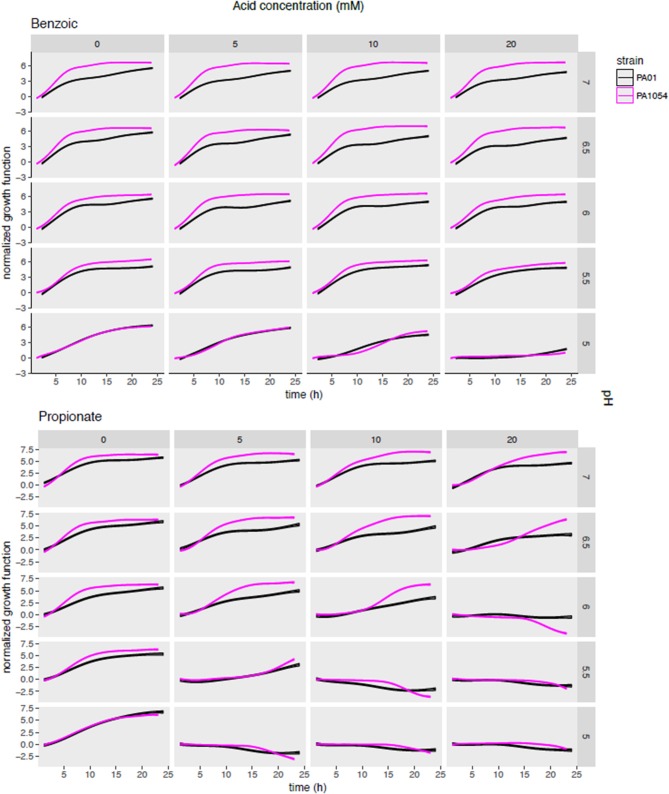
Posterior predictions of growth functions across OA/pH combinations and strains. Each curve represents the growth curve mean (center line of each curve) and standard deviation (width of curve) for phenom growth fit. Each graph depicts a growth curve for each OA/pH combination. Time in hours (0–25) is given on the X-axis and log2 normalized growth function (see Methods) given on the Y-axis. Individual axis numbers are not shown for clarity of the figure, but each sub-graph shown has the same axis ranges. OA concentrations are arranged across the columns of the graph grid, and pH levels down the rows. Black lines represent growth of PAO1 strain, magenta represents PA1054. Results from only two OA types are shown for clarity, with the results from the remaining acids shown in Figure S3.

To determine which OA/pH combinations were most effective in growth impairment for each of PAO1 and PA1054 strains, we first examined *phenom* model growth curve estimates (Figure 5, Supplementary Table 3). Each growth curve represents the mean of the model growth estimate given the data for a given OA/acid combination across all model terms combined (independent effects on growth for each of OA and pH alone, the effect of OA/pH interaction, and the grand mean, see Methods). Regardless of the OA type used, we observed that PA1054 grew faster and reached a higher carrying capacity than PAO1 when OA concentration was low and pH was near neutral, with the exception of malic acid (Figure 5, Figure S3). However, growth was abolished under the strongest conditions of pH 5 and 20 mM OA, an effect that appeared indistinguishable between the two strains. At high OA concentration and low pH, some acids were more effective in growth inhibition than others. For example, acetic acid at pH 5.5 was completely bacteriostatic, whereas benzoic acid at the same concentrations had no effect on growth (Figure 5, Figure S3).

### Across pH Levels, Some Organic Acids Have Significantly Stronger Bacteriostatic Effects Than Others Regardless of Strain Backgrounds

**Figure 6:**
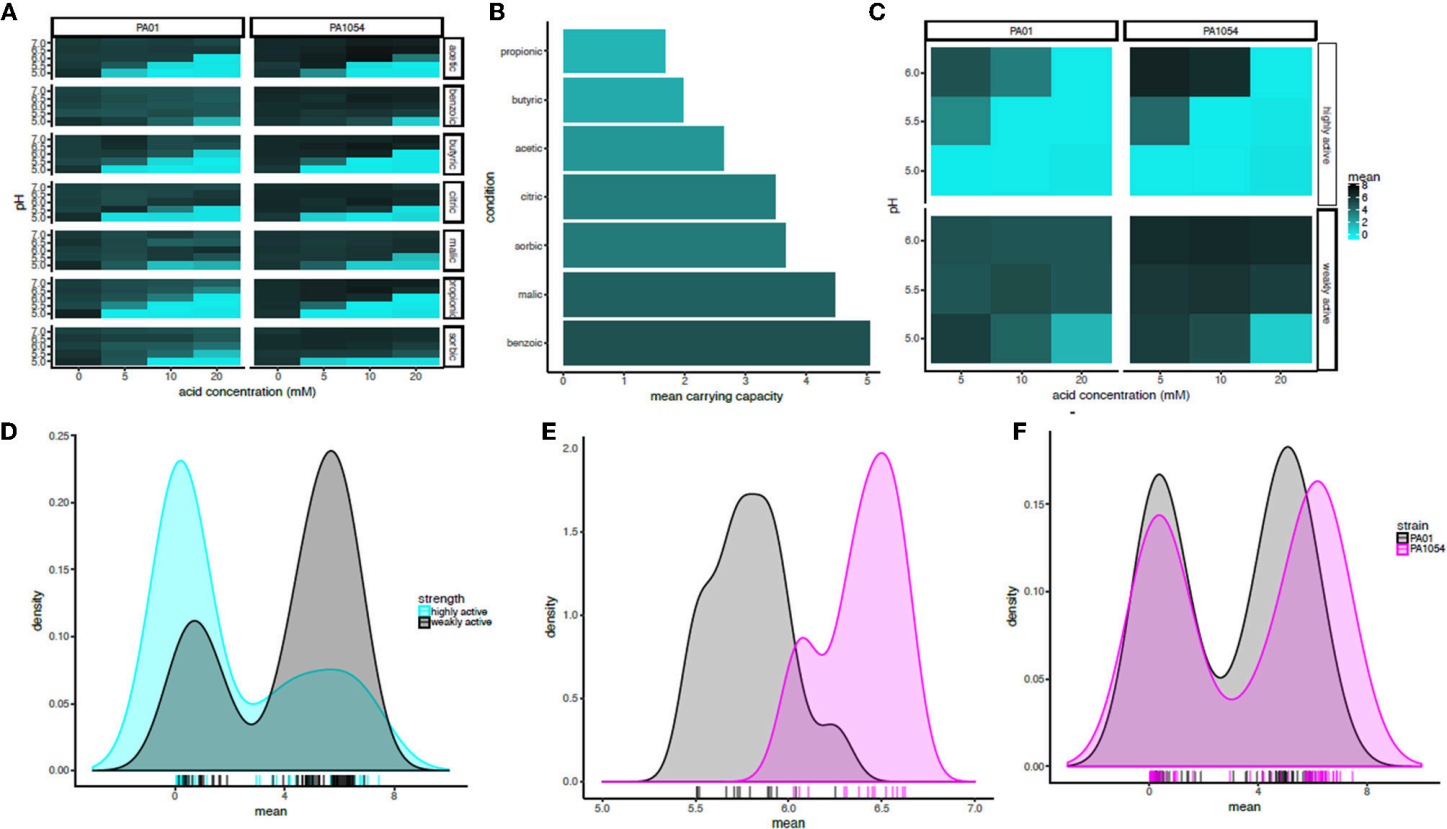
In combination with pH at or below 6, acetic, butyric, and propionic acid impair growth significantly more strongly than the other acids regardless of strain background. **(A)** Heat map of mean carrying capacity estimates from the phenom model. In each heat map subpanel, OA concentrations are shown on the X-axis and pH levels on the Y-axis. In the heat map grid, PAO1 strain is given in the left column and PA1054 in the right. OA type is given in the rows. Brighter cyan intensity indicates a lower carrying capacity (see color legend at far right). **(B)** Bar graph rank ordering OA types in terms of mean carrying capacity across strains. OA types with the greatest bacteriostatic effect are shown at top, the weakest on the bottom. **(C)** Heat map of the mean effect on carrying capacity for highly active OA types (propionic, butyric, acetic), vs. weakly active (citric, sorbic, malic, benzoic). Carrying capacities were averaged across acids in each category at each pH and across strains. **(D)** Density histogram of carrying capacities for highly active OAs (cyan) vs. weakly active OAs (gray) at growth limiting concentrations. Each tick in the rug plot beneath the histograms represents an individual carrying capacity value at a given pH/OA combination in a given strain. **(E)** Density histograms of carrying capacities for cultures of PAO1 and PA1054 at concentrations of OA and pH levels that allow for growth. Ticks in rug plot represent individual carrying capacity values. **(F)** Density histograms of carrying capacities for cultures of PAO1 (black) and PA1054 (magenta) at growth limiting concentrations. Ticks in rug plot represent individual carrying capacity values.

To quantify the statistical significance of the general trends observed by visual inspection of the growth function predictions from the model, we extracted carrying capacity estimates from the *phenom* model fits as a summary parameter (Supplementary Table 2). In general, the carrying capacity estimates reflect the observed trends in the differential effectiveness of OA/pH combinations on bacterial growth inhibition, suggesting that this is an informative summary metric for the growth estimates (compare Figure 5 with Figure 6A). This correspondence is in contrast to the commonly used growth parameter of maximum specific growth rate (μmax), which has no meaning for growth curves with no slope, such as those observed for cultures in high OA and low pH conditions. Carrying capacities of cultures grown in the different OA types were ranked according to the strength of the growth impairment, as quantified by the mean carrying capacity across low pH levels (≤6.0, acid concentration ≥5.0) and strains (Figure 6B). We found the ranking (from most active) to be as follows: propionic acid > butyric > acetic > citric > sorbic > malic > benzoic acid. We then categorized these OA types as “highly active” (propionic, butyric, acetic acids) or “weakly active” (citric, sorbic, malic, benzoic) based on this ranking. The order does not correlate with pKa or with whether the acid are mono-, di-, or tri-carboxylic. We chose to separate these categories between acetic and citric acid because the difference between the mean carrying capacities of these two OAs was larger than the difference between any other OAs that neighbor one another in the ranked list. Compared to weakly active OAs, cultures grown in highly active OAs had significantly lower carrying capacities in combination with low pH (pH ≤ 6, OA concentration ≥5 mM; *p* ≤ 1.121e-05; Figures 6C,D).

The quantitative effects of OA types on carrying capacities of PAO1 vs. PA1054 were consistent with our visual observations described above: in combinations of pH and OA that allowed for growth, PA1054 cultures reached significantly higher carrying capacities than those of PAO1 (*p*-value ≤ 5.096e-08; Figure 6E). In contrast, at growth limiting concentrations (OA ≥ 5 mM and pH ≤ 6), weakly active OAs appeared to be equally effective in limiting growth of PAO1 and PA1054 strains in combination with low pH (significance of carrying capacity differences between strains under growth limiting combinations for highly active OAs *p* ≤ 0.3858, weakly active OAs *p* ≤ 0.3968; Figure 6F). Taken together, these data show that, in terms of culture carrying capacity, and regardless of strain background, highly active OAs (propionic, butyric, acetic acids) may have more effective antimicrobial properties when combined with low pH than the other OAs tested here.

### Relative to Either pH or Acid Alone, the Combinatorial Effects of Propionic Acid × pH Is Significantly More Growth Inhibitory Than any Other OA/pH Combination

**Figure 7:**
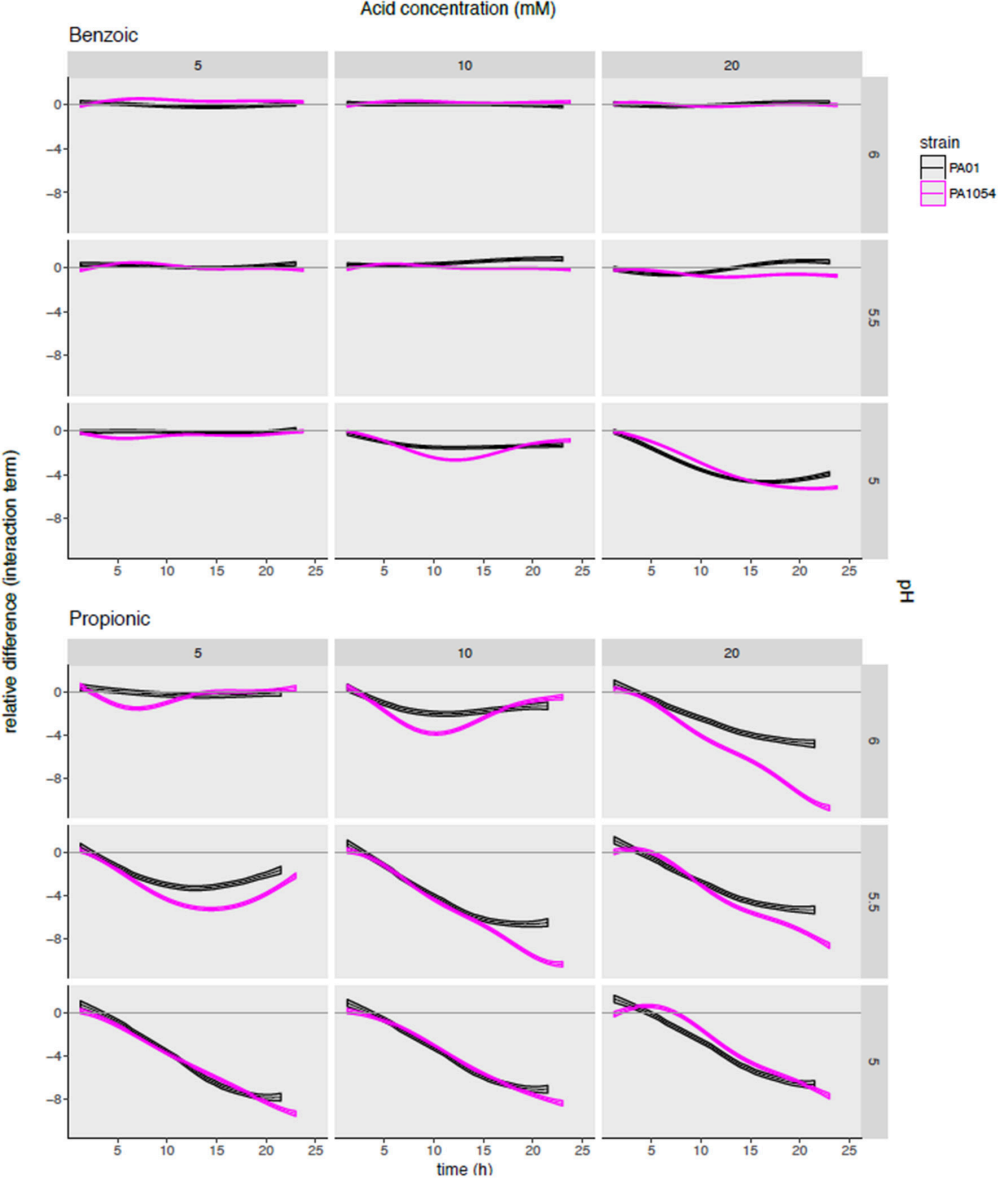
Modeling the combinatorial effects of OA/pH together relative to either pH or OA treatment alone pinpoints effective conditions specific for inhibiting growth of each strain. Each subgraph in the grid plots the posterior predictions of the interaction term functions across each OA/pH combination and strain. Each curve represents the function mean (center line of each curve) and standard deviation (width of curve) for phenom interaction term function. Time in hours (0–25) is given on the X-axis, and the difference in log2 normalized growth compared to either pH or OA independently [i.e., independent effect of pH, α_*p*_(*t*); and independent effect of OA, β_*m*_(*t*)] is given on the Y-axis. Individual axis numbers are not shown for clarity of the figure, but each sub-graph shown has the same axis ranges. OA concentrations are arranged across the columns of the graph grid, and pH levels down the rows. Black lines represent growth of PAO1 strain, magenta represents PA1054. Results from only two OA types (most highly active and most weakly active) are shown for clarity, with the results from the remaining acids shown in Figure S4.

To determine the synergistic effects on growth inhibition of combining OA and pH, and how this effect varies by strain and by OA type, we next examined the interaction term functions from the *phenom* model output, given by (αβ)_*p, m*_(*t*) (Methods, Supplementary Table 4). This function estimate captures the impact of the interaction between a given pH level and OA concentration relative to the independent effects of each of the two variables. We observed that the interaction term largely reflects the observations for carrying capacity in the sense that some OAs inhibit growth more effectively than others (Figure 7, Figure S3). For example, the only combination of conditions that were bacteriostatic for benzoic acid were at the highest acid concentration (20 mM) and lowest pH (5) (Figure 7).

The interaction term also reveals a synergistic effect on growth for certain OA × pH combinations that appeared different across the two strains. For example, malic acid at 20 mM + pH 5 and propionic acid at 20 mM, pH 6 appeared more effective in growth inhibition of PA1054 than PAO1 relative to either OA or pH alone (Figure S3). In contrast, 10 mM sorbic acid + pH 5.5 appeared more effective combination for inhibiting growth of PAO1 than PA1054.

**Figure 8:**
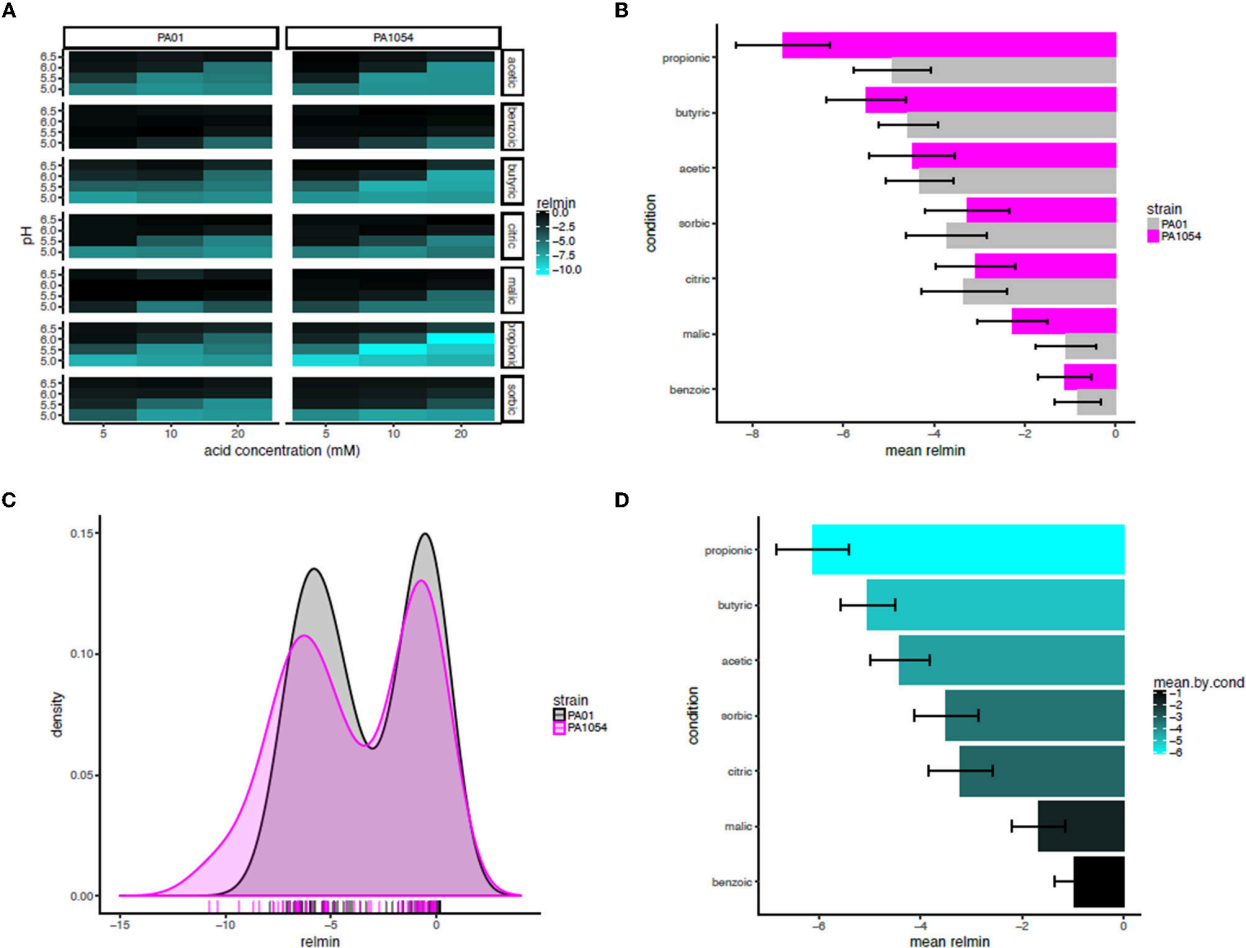
Propionic acid in combination with low pH is the most effective antimicrobial treatment for both strains. **(A)** Heat map of mean minimum relative difference (interaction term) estimates from the phenom model. In each heatmap subpanel, OA concentrations are shown on the X-axis and pH levels on the Y-axis. In the heatmap grid, PAO1 strain is given in the left column and PA1054 in the right. OA type is given in the rows, abbreviated as follows: ac., acetic; ben., benzoic; bu., butyric; cit., citric; mal., malic; prop., propionic; sor., sorbic. Brighter cyan intensity indicates a lower minimum interaction term estimate (see color legend at far right). **(B)** Bar graph rank ordering OA types in terms of mean relative minimum in each strain. OA types with the greatest bacteriostatic effect are shown at top, the ones with the least effect on the bottom. Gray bars, PAO1; magenta bars, PA1054. **(C)** Density histograms of minimum interaction terms for cultures of PAO1 and PA1054 at growth limiting concentrations of OA and pH levels. Ticks in rug plot represent individual carrying capacity values. **(D)** Bar graph rank ordering and quantifying mean of relmin for each OA across strains and OA types. Brighter cyan intensity indicates a lower minimum interaction term estimate (see color legend at far right).

Based on this observation, we used the minimum of the interaction term function (“relmin,” Supplementary Table 5) as a novel growth metric to summarize and calculate the statistical significance of the combinatorial effects of OA × pH, with the ultimate aim of determining the most effective combination(s) (Figure 8A). Consistent with our observations for the interaction term across the growth curve, according to relmin, the propionic acid × pH combination more strongly inhibited the growth of PA1054 than PAO1 (Figure 8A), although this difference was not significant (*p* ≤ 0.1743; Figure 8B). Similarly, the difference between strains was not significantly different on malic (*p* ≤ 0.409) or sorbic (*p* ≤ 0.7803) acids across low pH levels. Indeed, the behaviors of PAO1 and PA1054 were not significantly different at growth limiting combinations of OA and pH across all conditions according to relmin (0.2444, Figure 8C). However, when both strains were considered together, we observed that growth was more strongly and significantly inhibited by the combination of propionic acid and low pH compared to any other OA × pH combination (*p* ≤ 0.02209; Figure 8D). Thus, combinatorial effects modeling has enabled us to correct for differences in growth in each of pH or OA conditions alone, providing an advantage (which will be relevant to any similar studies) over classical growth metrics, such as carrying capacity. Moreover, using relmin as a growth metric pinpointed propionic acid × low pH as a more effective antimicrobial strategy than any other combination of conditions tested.

## Discussion

The treatment of infections with antimicrobial agents in addition to or in combination with antibiotics is likely to increase in coming years. Strategies that minimize unnecessary antibiotic use are needed because so many organisms are becoming more resistant to all types of antibiotics (World Health Organization., 2014). There is also an ongoing need for antibacterial treatments that are effective in parts of the world where advanced health care is not available or is too expensive. OAs have a history of extensive use in the food industry, where they have long been known to have antibacterial and antifungal properties. In this context, weak OAs [principally acetic acid, though work with citric acid has also been reported (Nagoba et al., 1998, 2013a)] have been used on a small scale for many years to treat infections of wounds, such as burn wounds, diabetic ulcers, and post-operative wounds (Phillips et al., 1968; Milner, 1992; Sloss et al., 1993; Nagoba et al., 1997, 2013b; Ryssel et al., 2009; Bjarnsholt et al., 2015; Halstead et al., 2015; Agrawal et al., 2017). However, most of these publications are in the clinical literature and generally report single cases or small surveys. The evolution of high levels of resistance to OAs has not been reported [though resistance to benzoate under mild acid conditions has been studied using laboratory-based evolution in *E. coli* (Creamer et al., 2017)], which may therefore be another advantage of the use of these agents to treat infections. As far as we are aware, effects of OA concentration and pH, and the interactions between them, have not been reported for *P. aeruginosa*, one of the most common opportunistic pathogens of infected wounds (Williams et al., 2009; Norbury et al., 2016). Indeed, information about the pH of the formulations used clinically is rarely given, although it can be calculated that many are used at a higher concentration and lower pH than we have used here (for example, a 5% (v/v) acetic acid solution, widely used in the literature cited above, will be 875 mM and have a pH of 2.91). This general lack of information prompted us to undertake the present study. Here, we have focussed on growth in a single defined medium and systematically evaluated the effectiveness of several OA types at stopping growth, including those widely used as food preservatives, paying particular attention to the interactions between pH and concentration. We have focused in this study on inhibition of growth rather than bactericidal action, which requires much higher concentrations of organic acids (FB, data not shown). Data on the effects of the different organic acids and conditions on biofilm formation and removal will be presented in a separate publication.

The combination of low pH and OAs are growth inhibitory for *P. aeruginosa*, as they are for many bacteria. Here we show that pH/OA treatment leads to growth behaviors which are very different from standard logistic growth, even at intermediate concentrations (Figures 1, 5, 7). In any study where stressors are being altered systematically, data about the impact of these changes should be captured so that comparisons are statistically robust, but our attempts to extract and compare growth parameters using logistic approaches were often not successful. The use of the Gaussian process regression-based method *phenom*, which makes no prior assumptions about the shape of the curve to which the data is being fitted, not only enabled us to generate estimates of growth parameters for all stress conditions, but also provided statistical measures of the significance of these conditions and the interactions between them. Under conditions of near to neutral pH and low or no added OA, parameters extracted using either approach correlated well, but our analysis clearly showed the superiority of *phenom* as stress levels increased and growth began to depart from logistic behavior. The different OAs varied in their effects on the two strains studied, with the laboratory strain PAO1 growing less well under non-stress conditions than the clinical isolate PA1054, but these differences disappeared as the stress levels increased. This observation implies that use of the laboratory strain to investigate effects of OA treatments on *P. aeruginosa* strains in general is statistically justified, although it would be desirable to extend this observation with a larger number of clinical isolates, such as exists in the international Pseudomonas reference panel (De Soyza et al., 2013). Among the OAs, acetic, propionic and butyric acid were all more inhibitory to growth than the other four acids tested (Figure 6).

One particular advantage of *phenom* compared to the previously developed B-GREAT method (Tonner et al., 2017) is that it provides an explicit decomposition of functional effects due to combinations of pH and OA as a function of time, thus enabling modeling of combinatorial effects on growth relative to either condition alone. This improves the B-GREAT method in two ways. First, the lengthscale of functional effects is estimated for each effect independently. This allows for greater flexibility in the shape of functions captured for each independent effect, while B-GREAT only considered a single, global lengthscale parameter. Second, by decomposing the functional effects into separate components, an explicit representation of the variance components is captured. This can be used for comparing overall impacts of independent experimental factors. B-GREAT had only a single variance hyperparameter for all effects combined. Our analysis showed that inhibition of growth increased both with increasing OA concentration and decreasing pH, and inspection of the summary metric relmin, which enables simple statistical tests on combinatorial effects, showed that the most significant interaction between these two factors was seen in the case of propionic acid (Figure 8). The unpleasant smell of propionic acid may restrict its use in the clinic, but we found for all the highly active OAs that strong inhibition of growth was seen at lower concentrations and higher pH levels than those normally reported in clinical studies. Whether these conditions are also able to have an impact on biofilm formation and eradication, and their relationship to levels that are bactericidal, will be the subject of a separate paper.

We have not, in this study, attempted to address the mechanisms by which the different OAs may be acting to prevent bacterial growth. The link between pH and the bacteriostatic effect of weak OAs has long been known, and is thought to arise in part from the ability of OAs to cross the membrane in their dissociated state, and to subsequently ionize in the bacterial cytoplasm (Eklund, 1983; Salmond et al., 1984; Russell, 1992; Russell and Diez-Gonzalez, 1998; Foster, 1999; Hirshfield et al., 2003; Saparov et al., 2006; Slonczewski et al., 2009). As the proportion of OA that is undissociated and able to enter the cell will increase as the pH drops, so this effect will become more pronounced as the pH of the growth medium is lowered. This may then cause decreased cell growth for a variety of reasons including depression of cytoplasmic pH, toxicity of the anion, osmotic stress, and partial collapse of the trans-membrane proton gradient (Brul and Coote, 1999; Hirshfield et al., 2003; Carpenter and Broadbent, 2009; Trček et al., 2015). The fact that the impact of OAs on growth is not solely caused by the decreased pH can be seen by the poor correlation of the carrying capacity with the pKa of the different organic acids (Table S1), an effect which has been noted previously (Sheu et al., 1975, Heavin et al., 2009; van Beilen et al., 2014). It should be noted that some of the toxic effects of organic acids can be attributed to their dissociated forms (Eklund, 1983), and that in the experiments described in this study, there will still be substantial amounts of dissociated OA present, as even the lowest pH used is well above the pKa for any of the acids. In addition, OAs partition into membranes to different extents dependent on the nature of their aliphatic regions, which can in turn disrupt membrane function (Alakomi et al., 2000; van Beilen et al., 2014). But as our results here show, these partition coefficients for the different OAs also show no correlation with the impact of the OAs on growth (see Table S1). The poor ability of sorbic and benzoic acids to reduce *P. aeruginosa* growth was of particular interest given their widespread and effective use as food preservatives, but the mechanistic reasons for this remain to be explored. The overall impact of different OAs on growth presumably arises from the interaction of many effects, and in the absence of a complete understanding or a predictive model of these, experimental assessment on a case-by-case basis where the impacts of multiple parameters and the interactions between them are tested experimentally is still the best method to determine whether a given OA is a suitable choice for any given application.

This study establishes a reliable method for detecting such effects even when growth curves cannot be fitted by logistic models. This will be particularly useful in looking at the effects of combinations of different anti-microbial compounds at a range of dosage levels, as is now possible using automated high-throughput methods (Chevereau and Bollenbach, 2015; Brochado et al., 2018). Indeed, the use of approaches, such as the *phenom* method described here has considerable potential in any studies concerned with developing new treatments where combinations of compounds are used, as methods to robustly analyse drug combinations have long been sought (Greco et al., 1995; Twarog et al., 2016). The availability of a method that does not fail when growth behavior departs from standard models, and which can statistically account for the strength of interactions between multiple variables, will help searches of large “phenomics” experiments in which conditions studied will be clinically highly relevant, while also aiding the development of a deeper mechanistic understanding of the biological processes underlying the impacts of different stressors on growth.

## Author Contributions

PL and FB conceived the original study and designed the experiments. FB generated all the experimental data. FB, PL, AS, PT, and SJ analyzed the experimental data. FB, PL, AS, PT, and SJ wrote the paper.

### Conflict of Interest Statement

The authors declare that the research was conducted in the absence of any commercial or financial relationships that could be construed as a potential conflict of interest.
