# Supplementary material for: Synergistic Impacts of Organic Acids and pH on Growth of Pseudomonas aeruginosa: A Comparison of Parametric and Bayesian Non-parametric Methods to Model Growth
Source: Front Microbiol. 2019 Jan 8;9:3196. doi: 10.3389/fmicb.2018.03196 (PMC6331447; doi:10.3389/fmicb.2018.03196)
Supplement: Supplementary file 6 [file Data_Sheet_1.docx]

Supplementary Figures

**Figure S1: Growth curve of *P. aeruginosa* strain PAO1 in supplemented buffered M9 at pH 7.** Data points are averaged from 60 wells across a 96 well plate, error bars show standard errors of the mean. No growth was monitored in columns or rows at the edge of the plate, as this was found to increase variability.


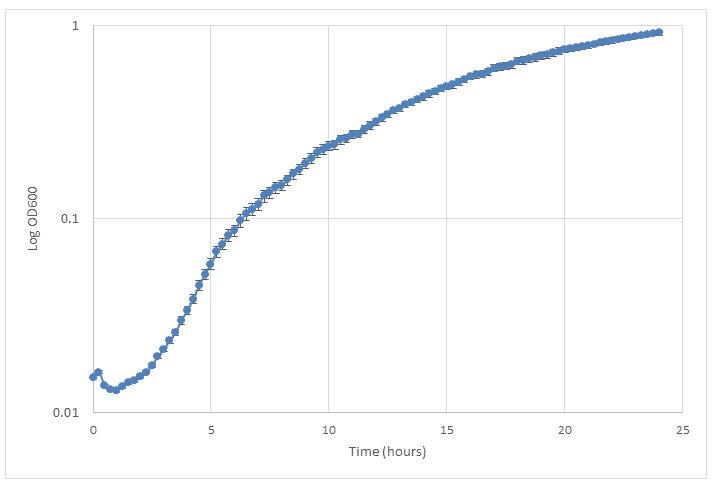


**Figure S2: Growth curve of *P. aeruginosa* strain PAO1 in supplemented buffered M9 at pH 7 to pH 5 plus 10 mM organic acid.** Data points are averages from 6 independent replicates, error bars show standard errors of the mean.

A

B

C

D

E

**Figure S3: Posterior predictions of growth functions across OA / pH combinations and strains.** These are all the cases of which two representative examples are shown in Figure 5. The descriptive legend for this figure is the same as for Figure 5.


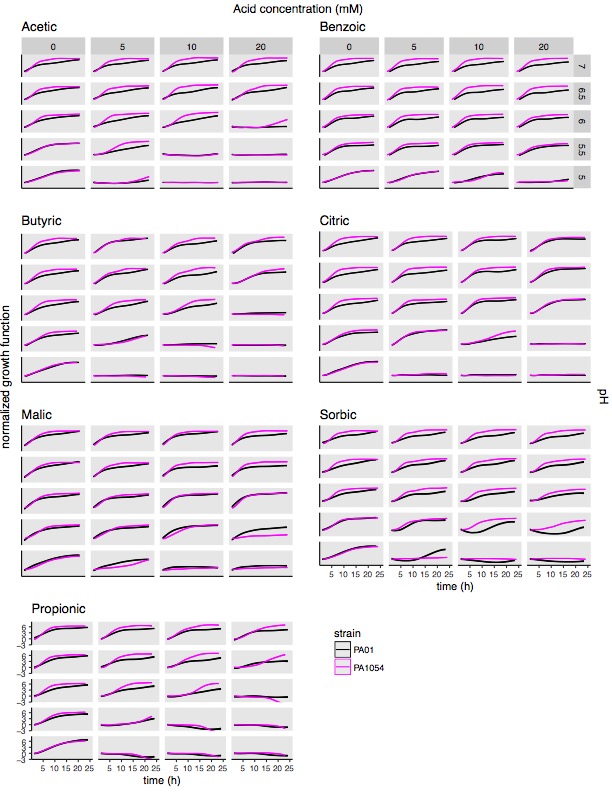


**Figure S4. Modelling the combinatorial effects of OA / pH together relative to either pH or OA treatment alone pinpoints effective conditions specific for inhibiting growth of each strain.** These are all the cases of which two representative examples are shown in Figure 7. The descriptive legend for this figure is the same as for Figure 7.


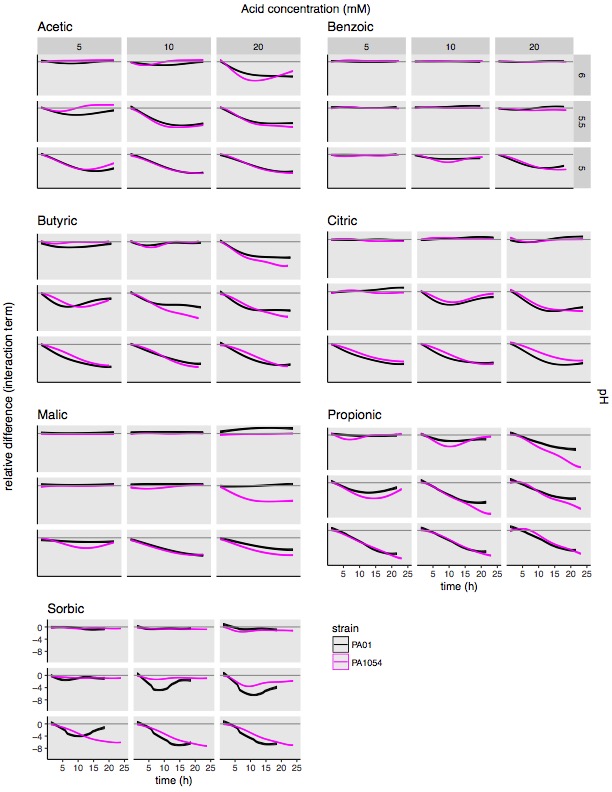


**Table S1:** Organic acids in this studied ranked by their impact on carrying capacity, and their respective pKa and logP_ow_ values.

| Organic acid | Impact on mean carrying capacity (*phenom*) from high (1) to low (7) | pKa value | Number of ionisable carboxyl groups | Partition co-efficient (log P_ow_, octanol/water) |
| --- | --- | --- | --- | --- |
| Propionic | 1 | 4.87^1^ | 1 | 0.33^3^ |
| Butyric | 2 | 4.82^1^ | 1 | 0.79^3^ |
| Acetic | 3 | 4.74^1^ | 1 | -0.17^3^ |
| Citric | 4 | 3.13, 4.76, 6.4^2^ | 3 | -1.7^3^ |
| Sorbic | 5 | 4.76^1^ | 1 | 1.33^3^ |
| Malic | 6 | 3.51, 5.03^1^ | 2 | -1.26^4^ |
| Benzoic | 7 | 4.19^1^ | 1 | 1.87^3^ |

Data from ^1^pubchem (<https://pubchem.ncbi.nlm.nih.gov/>); ^2^Merck index online (<https://www.rsc.org/Merck-Index/>); ^3^Inchem (<http://www.inchem.org/>); ^4^ECHA (https://echa.europa.eu/)
